# Supplementary material for: Community engagement in mass drug administration participatory interventions: A scoping review
Source: PLoS Negl Trop Dis. 2025 Dec 1;19(12):e0013737. doi: 10.1371/journal.pntd.0013737 (PMC12742743; doi:10.1371/journal.pntd.0013737)
Supplement: S1 Text — (PDF) [file pntd.0013737.s001.pdf]

May 22, 2025

Community engagement in mass drug administration participatory interventions: a scoping review

DOI
dx.doi.org/10.17504/protocols.io.8epv5xq15g1b/v1

Anouk Chouaïd[1]
[1]Université Paris Cité

Scoping review commun...

Anouk Chouaïd
Université Paris Cité

OPEN ACCESS

DOI: dx.doi.org/10.17504/protocols.io.8epv5xq15g1b/v1

Protocol Citation: Anouk Chouaïd 2025. Community engagement in mass drug administration participatory interventions: a scoping review. **protocols.io** https://dx.doi.org/10.17504/protocols.io.8epv5xq15g1b/v1

License: This is an open access protocol distributed under the terms of the **Creative Commons Attribution License**, which permits unrestricted use, distribution, and reproduction in any medium, provided the original author and source are credited

Protocol status: Working
We use this protocol and it's working

Created: January 23, 2024

Last Modified: May 22, 2025

Protocol Integer ID: 93953

Abstract

Introduction:

Over the past decade, mass drug administration (MDA) has received increasing attention in the context of infectious disease control strategies(1). It is most often part of the therapeutic arsenal for malaria eradication and management of neglected tropical diseases. The condition for the effectiveness of such an intervention is the participation of the population and therefore the support of the community. Moreover, community engagement (CE) is increasingly recognized as a key element of health research because of its contribution to more ethical, relevant, and well-conducted research. (2) While CE activities are increasingly implemented, there is a lack of clarity about the purposes of engagement, its implementation challenge, and its evaluation.

Objective:

Several studies and reviews of mass administration ofantimalarial drugs mention community involvement initiatives (3,4), but don't always describe and evaluate the effect of community participation. A realist review published in 2016 aimed to synthetise evidence prior to 2013. (5) It called for more systematic evaluation of engagement. The number of articles describing mass drug administration initiatives has grown exponentially since then and the use of participatory community engagement interventions is growing, making it necessary to update knowledge on the subject. The purpose

of this scoping review is to identify how community engagement was mobilized during participatory interventions implemented for mass drug administration studies. This review is conducted to inform a population health intervention research project of mass administration of antimalarials in a rural region of Senegal. The research project will have a participatory approach and will involve the co-construction of the intervention design with the populations concerned. We have chosen the scoping review approach because this review is an initial exploratory study that aims to provide an overview of the knowledge about community involvement in participatory intervention on mass drug administration and to map the available evidence in this area.

Inclusion criteria:

To be eligible, an article has to be peer-reviewed, be an empirical paper, written in English or French. Its full text must be available. Articles describing participatory interventions about mass drug administration will be included. Abstracts without the full text available, commentaries, editorials and conceptual/theoretical papers will be excluded.

Methods:

The search strategy for the bibliographic databases will be developed by the research team in collaboration with experienced researchers. We will search on PubMed, Embase, Cochrane library, Scopus, and Web of Science for papers published, supplemented by expert bibliography and references cited in the documents analysed. The research will cover studies published in French and English and up to December 2023 will be included. The review and extraction of references (abstracts and full articles) will be carried out by the research team using Covidence. The data will be analysed by the lead author according to an analytical grid based on the categorisation of the type of intervention, the model used for its design or implementation, the stakeholders and their modes of involvement, the limiting and facilitating factors, the evaluation of the

intervention, its sustainability and replicability, and the conclusions and recommendations/lessons learned of the authors.

Title

1 **Community engagement in mass drug administration participatory interventions: a scoping review**

Authorship

2 A. Chouaïd, S. Louart, E. Bah, J. Landier, V. Ridde

Introduction

3 MDA is the practice of administering a drug treatment to the entire population in a given area, regardless of knowledge of symptoms or presence of infection (1). MDA is carried out in a coordinated manner so that the drug is taken at the same time by the entire population. The objectives of MDA can be to reduce or interrupt transmission, to reduce morbidity and mortality, or to prevent relapse and resultant infectious transmission. The colonial history of MDA intervention shows just how important it is to consider the social context and the power issues involved in its deployment. (6,7) MDA interventions had different fates after the colonial era. MDA persisted as a key intervention in the control and elimination of neglected tropical diseases such as onchocerciasis, dracunculiasis, lymphatic filariasis (mainly worms) but also vitamin A distribution... On the other hand, it was largely abandoned from the malaria portfolio for several decades, between the 1980s and the 2000s. During that period, multiple antimalarial drugs fell to resistance.

The condition for the effectiveness of such an intervention is the comprehensive participation of the population and therefore the support of the community. Community-based participatory (CBP) intervention theory suggests that engaging community members as collaborators in the interventions to reduce health disparities is powerful on multiple levels (8). Community engagement (CE) is increasingly recognized as a key element of health research because of its contribution to more ethical, relevant, and well-conducted research. CE has been defined as a process of working collaboratively with groups of people linked by geography, interest or health issues to address social and health challenges affecting them (9). Research ethics guidelines now often include significant involvement of research participants at all steps of the research process. (9)(10)

While CE activities related to health research have proliferated, particularly in low- and middle-income countries (LMICs), and are increasingly described in the literature, there is a lack of clarity about the goals and purposes of engagement, its practical

implementation challenges, and its evaluation. For example, the distinction between instrumental (improve the quality of the research) and ethical goals in often unclear (11). Ultimately, the evidence for engagement remains underdeveloped.
The purpose of this scoping review is to identify how community engagement was mobilized during participatory interventions implemented for MDA studies. This review is conducted to inform a population health intervention research of mass administration of antimalarials in a rural region of Senegal. The research project will have a participatory approach and will involve the co-construction of the intervention theory with the populations concerned.
Several studies and reviews of mass administration of antimalarial drugs mention community involvement initiatives (3,4). The concept of community participation is not used in the same way by all the authors, and the involvement of research participants in the different steps of the research process is not always described and evaluated. A systematic review published in 2016 and including articles published prior to 2013 aimed to describe community involvement in mass antimalarial drug administration interventions. (5) It called for more systematic evaluation of engagement. The number of articles describing mass drug administration initiatives has grown exponentially in the last decade and the use of participatory community engagement interventions is growing, making it necessary to update knowledge on the subject.

Review question

4 What is known about community engagement in participatory interventions for mass drug administrations?
Sub-questions (specific objectives):
- What are authors referring to when they use the concept of community engagement? How is community engagement defined and what kind of activities are implemented to ensure community involvement?
- What theories, frameworks or models were used to inform the design or content of the interventions for community engagement?
- What strategies were used to evaluate community engagement?
- What factors helped or hindered community engagement when these interventions were implemented?
- What conclusions and recommendations have been drawn from these interventions?

Keywords

5 Search terms, developed with an experience researcher to capture relevant literature, included our three concepts of interest as described below.

A	B
**Community engagement**	**Mass drug administration**

A	B
Community-based intervention, Community-Based Participatory Research	Mass drug administration
Action research, Community action	MDA
Community engagement, participation, partnership, collaboration, involvement, consultation, meeting, mobilization, empowerment, representative, input, led, driven	Mass chemoprophylaxis
Stakeholder engagement, participation, partnership, collaboration, involvement, consultation, meeting	Mass administration
Public consultation, meeting, mobilisation	Mass distribution
User involvement, participation, contribution	Mass treatment
Consumer participation, engagement, involvement, driven, consultation, empowerment	Mass therapeutic
Citizen engagement, participation, involvement, deliberation, consultation, empowerment	Coordinated administration, distribution, treatment
Patient participation, involvement, contribution, consultation	

Table 1 : Search terms

Eligibility criteria

6

7 · Participants Our review considers community engagement in the broadest sense, rather than a specific population.

8 · Concept The scope of the review is on community engagement and mass drug administration. All papers that do not include these two concepts will be excluded. The scoping aims to cover the literature about mass drug administration intervention, which is the administration of a treatment irrespective of the knowledge of symptoms or presence of infection, to an entire population in a given area. It will not include interventions of mass screening and treatment (MSAT) and focal screening and treatment (FSAT), that require testing all people in a geographical area and treating only positive cases. Likewise, it will not include mass interventions targeting a specific population subgroup, such as seasonal malaria chemoprevention, which mass drug administration restricted to children under 5 (or 10) years. Interventions about focal MDA (also called focal drug administration or targeted MDA), which refers to reactive interventions triggered by a clinical case and propose a treatment to asymptomatic persons around the infected one, will not be included either. Our review focuses on community engagement in participatory interventions. Articles dealing with an intervention described by its authors as participatory will be included. Moreover, an intervention not initially described as participatory that became so avec the course of the implementation process will also be included. Within these articles, the notion of community engagement must also necessarily be evoked. We adopted a very broad definition of community involvement, to include all articles that mention initiatives that could be linked to it (see Appendix for definitions). Based on the assumption that there is a paucity of scientific literature on this, we decided not to limit the review to references that evaluated the effectiveness of the interventions. Interesting references that do not include an evaluation of the strategy implemented may be included and discussed with the authors at a later stage if relevant.

9 · Context This review covers all geographical areas and does not have a time limit.

10 · Type of sources This review will consider qualitative, quantitative and mixed-methods studies reported in peer-reviewed empirical publications. Systematic reviews will not be included as such, but their list of references/articles will be searched to find potential studies to include. Abstracts, commentaries, and editorials will be excluded. Experimental and quasi-experimental interventions will be considered.

Methods

11 The proposed scoping review will be conducted in accordance with JBI's methodology for scoping reviews (12). We have chosen the scoping review approach because this review is an initial exploratory study that aims to provide an overview of the knowledge

about community involvement in participatory intervention on mass drug administration and to map the available evidence in this area.

12 · Search strategy

The search strategy is developed by the research team, including researchers qualified in scoping reviews. It aims to locate only published studies. An initial limited search of PUBMED was undertaken to identify articles on the topic. The text words contained in the titles and abstracts of relevant articles, and the index terms used to describe the articles were used to develop a full search strategy for PUBMED (see Appendix). Additionally, scoping reviews on community involvement or MDA were studied and their search equations have been used to refine our own. We will search on PubMed, Embase, Cochrane library, Global index medicus, Scopus, and Web of Science for papers published. These databases were selected based on the subject areas covered by the indexed references (biomedical sciences for PubMed, Embase, Global index medicus and Cochrane library, transdisciplinary for Scopus and Web of Science) and the quality of the indexing and the references. The search strategy, including all identified keywords and index terms, will be adapted for each included database and/or information source. The reference list of all included sources of evidence will be screened for additional studies. Studies published in French and English and up to December 2023 will be included.

The full search strategy and selection criteria are presented in the appendix.

13 · Study/Source of Evidence selection

Following the search, all identified citations will be collected and imported into Zotero 6.0.12 (AGPL) ©, then uploaded to Covidence where duplicates will be removed. Titles and abstracts will then be screened by two independent reviewers for assessment against the inclusion criteria for the review. Potentially relevant sources will be retrieved in full and their citation details. The full text of selected citations will be assessed in detail against the inclusion criteria by two independent reviewers.

Reasons for exclusion of sources of evidence in full text that do not meet the inclusion criteria will be recorded and reported in the scoping review. Any disagreements that arise between the reviewers at each stage of the selection process will be resolved through discussion, or with an additional reviewer. The results of the search and the study inclusion process will be fully reported in the final scoping review and presented in a Preferred Reporting Items for Systematic Reviews and Meta-analyses extension for scoping review (PRISMA-ScR) flow diagram.

14 · Data extraction

Data will be extracted from the papers included in the scoping review by the lead reviewer

The data extracted will include specific details on :

(i) Author/s; Name of journal; Year of publication; Title;
(ii) intervention name/label;
(iii) geography and length of the intervention;
(iv) description of the mass administration intervention
(v) assessment of the methodological quality with the Mixed Methods Appraisal Tool (MMAT), version 2018
(vi) definition of community involvement given by the authors / directors
(vii) completeness of reporting and replicability of the interventions in favor of community engagement with TIDieR (Template for Intervention Description and Replication) (13)
- rationale, theory, and goals of the intervention
- description of the procedures, activities, processes used I the intervention
- description of the modes of delivery, the number of times the intervention was delivered and whether it was provided individually or in a group
- description of any adaptation or personalization of the intervention
- description of the adherence to the planned intervention

(viii) limiting and facilitating factors of community involment,
~~(ix)~~ stakeholder involvement (who and how);
(x) recommendations and conclusions.

The draft data extraction tool will be modified and revised as necessary during the process of extracting data from each included evidence source. Modifications will be detailed in the scoping review. Any disagreements that arise between the reviewers will be resolved through discussion, or with an additional reviewer. Where appropriate, authors of papers will be contacted to request missing or additional data as necessary.

Conflict of interest

15 There is no conflict of interest in this project.

Appendix

16

A	B
**Concept**	**Definitions and meaning**
Community engagement	Involvement of members of the community in the affairs of that community. We are referring to the

A	B
	range of strategies undertaken alongside research, for example meetings and discussions with the stakeholders, and training and devolvement of responsibilities to community volunteers. (9)
Mass drug administration	Administration of a medication to at-risk individuals in a population without individual diagnosis. It is often used in order to treat, control, and/or prevent spread of often endemic diseases outbreaks in high disease burden areas(1).

Table 2. Definition and meaning of concepts

17

A	B	C
	**Community engagement**	**Mass drug administration**
**Free words**	Community-based intervention*<br>participatory action Research<br>participatory research<br>participatory engagement*<br>community research<br>action research<br>Community-Based Participatory Research<br>Community engagement*<br>Community participation*<br>Community partnership<br>Community collaboration*<br>Community involvement*<br>Community consultation*<br>Community meeting*<br>Community mobilization*<br>Community empowerment<br>Community representative<br>Community input<br>Community led<br>Community driven<br>Stakeholder engagement*<br>Stakeholder participation*<br>Stakeholder partnership<br>Stakeholder collaboration*<br>Stakeholder involvement*<br>Stakeholder consultation*<br>Stakeholder meeting*<br>Public consultation*<br>Public meeting*<br>Public mobilization*<br>Public participation*<br>User involvement<br>User participation	Mass drug administration*<br>MDA<br>Mass chemoprophylaxis<br>Mass administration*<br>Mass distribution*<br>Mass treatment*<br>Mass therap*<br>Coordinated administration*<br>Coordinated distribution*<br>Coordinated treatment*

A	B	C
	User contribution<br>Consumer participation<br>Consumer engagement<br>Consumer involvement<br>Consumer driven<br>Consumer consultation<br>Consumer empowerment<br>Citizen engagement<br>Citizen participation<br>Citizen involvement<br>Citizen deliberation<br>Citizen consultation<br>Citizen empowerment<br>Community action*<br>Patient participation<br>Patient involvement<br>Patient consultation<br>Patient contribution	
**Thesaurus /database**	Medline :<br><br>Stakeholder participation<br>Community participation<br>Community-Based Participatory Research	Medline :<br>Mass drug administration
Embase :<br><br>stakeholder<br><br>engagement	Embase :<br>Mass drug administration	
Cochrane :<br><br>Stakeholder<br><br>participation<br><br>Community<br><br>participation	Cochrane :<br>Mass drug administration	
	global index medicus :<br>Community-Based	global index medicus :<br>Mass

A	B	C
	Participatory Research **Community Participation** **Stakeholder Participation**	drug administration

Table 3. Search strategy

18 EMBASE

'mass drug administration'/exp OR 'mass drug administration':ab,ti OR 'mda':ab,ti OR 'mass chemoprophylaxis':ab,ti OR 'mass administration':ab,ti OR 'mass distribution':ab,ti OR 'mass treatment':ab,ti OR 'mass therap*':ab,ti OR 'coordinated administration':ab,ti OR 'coordinated distribution':ab,ti OR 'coordinated treatment':ab,ti

AND

'stakeholder engagement'/exp OR 'community-based intervention':ab,ti OR 'participatory action research':ab,ti OR 'participatory research':ab,ti OR 'participatory engagement':ab,ti OR 'community research':ab,ti OR 'action research':ab,ti OR 'community-based participatory research':ab,ti OR 'community engagement':ab,ti OR 'community partnership':ab,ti OR 'community participation':ab,ti OR 'community collaboration':ab,ti OR 'community involvement':ab,ti OR 'community consultation':ab,ti OR 'community meeting':ab,ti OR 'community mobilization':ab,ti OR 'community empowerment':ab,ti OR 'community representative':ab,ti OR 'community input':ab,ti OR 'community led':ab,ti OR 'community driven':ab,ti OR 'stakeholder engagement':ab,ti OR 'stakeholder participation':ab,ti OR 'stakeholder partnership':ab,ti OR 'stakeholder collaboration':ab,ti OR 'stakeholder involvement':ab,ti OR 'stakeholder consultation':ab,ti OR 'stakeholder meeting':ab,ti OR 'public consultation':ab,ti OR 'public meeting':ab,ti OR 'public mobilization':ab,ti OR 'public participation':ab,ti OR 'user involvement':ab,ti OR 'user participation':ab,ti OR 'user contribution':ab,ti OR 'consumer participation':ab,ti OR 'consumer engagement':ab,ti OR 'consumer involvement':ab,ti OR 'consumer driven':ab,ti OR 'consumer consultation':ab,ti OR 'consumer empowerment':ab,ti OR 'citizen engagement':ab,ti OR 'citizen participation':ab,ti OR 'citizen involvement':ab,ti OR 'citizen deliberation':ab,ti OR 'citizen consultation':ab,ti OR 'citizen empowerment':ab,ti OR 'community action':ab,ti OR 'patient participation':ab,ti OR 'patient involvement':ab,ti OR 'patient consultation':ab,ti OR 'patient contribution':ab,ti

19 Pubmed equation :

"community participation"[MeSH Terms] OR "stakeholder participation"[MeSH Terms] OR "community based participatory research"[MeSH Terms] OR "community based

intervention*"[Title/Abstract] OR "participatory action research"[Title/Abstract] OR "participatory research"[Title/Abstract] OR "participatory engagement*"[Title/Abstract] OR "community research"[Title/Abstract] OR "action research"[Title/Abstract] OR "community based participatory research"[Title/Abstract] OR "community engagement*"[Title/Abstract] OR "community participation*"[Title/Abstract] OR "community partnership*"[Title/Abstract] OR "community consultation*"[Title/Abstract] OR "community meeting*"[Title/Abstract] OR "community mobilisation*"[Title/Abstract] OR "community empowerment"[Title/Abstract] OR "stakeholder engagement*"[Title/Abstract] OR "stakeholder participation*"[Title/Abstract] OR "Stakeholder partnership"[Title/Abstract] OR "stakeholder collaboration*"[Title/Abstract] OR "stakeholder involvement*"[Title/Abstract] OR "stakeholder consultation*"[Title/Abstract] OR "stakeholder meeting*"[Title/Abstract] OR "public consultation*"[Title/Abstract] OR "public meeting*"[Title/Abstract] OR "public mobilisation*"[Title/Abstract] OR "public participation*"[Title/Abstract] OR "local meeting*"[Title/Abstract] OR "Local empowerment"[Title/Abstract] OR "community action*"[Title/Abstract] OR "Community representative"[Title/Abstract] OR "Community input"[Title/Abstract] OR "Community led"[Title/Abstract] OR "Community driven"[Title/Abstract] OR "User involvement"[Title/Abstract] OR "User participation"[Title/Abstract] OR "User contribution"[Title/Abstract] OR "Consumer participation"[Title/Abstract] OR "Consumer engagement"[Title/Abstract] OR "Consumer involvement"[Title/Abstract] OR "Consumer driven"[Title/Abstract] OR "Consumer consultation"[Title/Abstract] OR "Consumer empowerment"[Title/Abstract] OR "Citizen engagement"[Title/Abstract] OR "Citizen participation"[Title/Abstract] OR "Citizen involvement"[Title/Abstract] OR "Citizen deliberation"[Title/Abstract] OR "Citizen consultation"[Title/Abstract] OR "Citizen empowerment"[Title/Abstract] OR "patient participation"[Title/Abstract] OR "patient involvement"[Title/Abstract] OR "patient consultation"[Title/Abstract] OR "patient contribution"[Title/Abstract]

20 **WEB OF SCIENCE :**
ALL=("mass drug administration") OR ALL=(MDA) OR ALL=("Mass chemoprophylaxis") OR ALL=("Mass administration") OR ALL=("Mass distribution") OR ALL=("Mass treatment") OR ALL=("Mass therap*") OR ALL=("Coordinated administration") OR ALL=("Coordinated distribution") OR ALL=("Coordinated treatment")
AND
ALL=("Community-based intervention") OR ALL=("participatory action Research") OR ALL=("participatory research") OR ALL=("participatory engagement") OR ALL=("community research") OR ALL=("action research") OR ALL=("Community-Based Participatory Research") OR ALL=("Community engagement") OR ALL=("Community participation") OR ALL=("Community partnership") OR

ALL=("Community collaboration") OR ALL=("Community involvement") OR ALL=("Community consultation") OR ALL=("Community meeting") OR ALL=("Community mobilization") OR ALL=("Community empowerment") OR ALL=("Community representative") OR ALL=("Community input") OR ALL=("Community led") OR ALL=("Community driven") OR ALL=("Stakeholder engagement") OR ALL=("Stakeholder participation") OR ALL=("Stakeholder partnership") OR ALL=("Stakeholder collaboration") OR ALL=("Stakeholder involvement") OR ALL=("Stakeholder consultation") OR ALL=("Stakeholder meeting") OR ALL=("Public consultation") OR ALL=("Public meeting") OR ALL=("Public participation") OR ALL=("User involvement") OR ALL=("User participation") OR ALL=("User contribution ") OR ALL=("Consumer participation ") OR ALL=("Consumer engagement ") OR ALL=("Consumer involvement") OR ALL=("Consumer driven") OR ALL=("Consumer consultation") OR ALL=("Consumer empowerment") OR ALL=("Citizen engagement") OR ALL=("Citizen participation") OR ALL=("Citizen involvement") OR ALL=("Citizen deliberation") OR ALL=("Citizen consultation") OR ALL=("Citizen empowerment") OR ALL=("Community action") OR ALL=("Patient participation") OR ALL=("Patient involvement") OR ALL=("Patient consultation") OR ALL=("Patient contribution")

21 cochrane library
Search Name:
Date Run: 05/01/2024
08:57:44
Comment:

ID Search Hits
#1 MeSH
descriptor: [Mass Drug Administration] explode all trees 155
#2 ("mass
drug administration"):ti,ab,kw OR ("MDA"):ti,ab,kw OR
("mass chemoprophylaxis"):ti,ab,kw OR ("mass
administration"):ti,ab,kw OR ("mass distribution"):ti,ab,kw 4519
#3 ("Mass
treatment"):ti,ab,kw OR ("coordinated administration"):ti,ab,kw
OR ("coordinated distribution"):ti,ab,kw OR ("coordinated
treatment"):ti,ab,kw 197

#4 #1 OR #2 OR
#3 4683
#5 MeSH
descriptor: [Stakeholder Participation] explode all trees 210
#6 MeSH
descriptor: [Community Participation] explode all trees 2481
#7 ("Community-based
intervention"):ti,ab,kw OR ("participatory action
Research"):ti,ab,kw OR ("participatory research"):ti,ab,kw OR
("participatory engagement"):ti,ab,kw OR ("community
research"):ti,ab,kw 1611
#8 ("action
research"):ti,ab,kw OR ("Community-Based Participatory
Research"):ti,ab,kw OR ("Community engagement"):ti,ab,kw OR
("Community participation"):ti,ab,kw OR ("Community
partnership"):ti,ab,kw 2705
#9 ("Community
empowerment"):ti,ab,kw OR ("Community representative"):ti,ab,kw
OR ("Community input"):ti,ab,kw OR ("Community
led"):ti,ab,kw OR ("Community driven"):ti,ab,kw 250
#10 ("Stakeholder
engagement"):ti,ab,kw OR ("Stakeholder participation"):ti,ab,kw
OR ("Stakeholder partnership"):ti,ab,kw OR ("Stakeholder
collaboration"):ti,ab,kw OR ("Stakeholder involvement"):ti,ab,kw 322
#11 ("Stakeholder
consultation"):ti,ab,kw OR ("Stakeholder meeting"):ti,ab,kw OR
("Public consultation"):ti,ab,kw OR ("Public
meeting"):ti,ab,kw OR ("Public mobilization"):ti,ab,kw 37
#12 ("Public
participation"):ti,ab,kw OR ("User involvement"):ti,ab,kw OR
("User participation"):ti,ab,kw OR ("User
contribution"):ti,ab,kw OR ("Consumer participation"):ti,ab,kw 209
#13 ("Consumer
engagement"):ti,ab,kw OR ("Consumer involvement"):ti,ab,kw OR
("Consumer driven"):ti,ab,kw OR ("Consumer
consultation"):ti,ab,kw OR ("Consumer empowerment"):ti,ab,kw 73
#14 ("Citizen
engagement"):ti,ab,kw OR ("Citizen participation"):ti,ab,kw OR
("Citizen involvement"):ti,ab,kw OR ("Citizen
deliberation"):ti,ab,kw OR ("Citizen consultation"):ti,ab,kw 6
#15 ("Citizen
empowerment"):ti,ab,kw OR ("Community action"):ti,ab,kw OR

("Patient participation"):ti,ab,kw OR ("Patient involvement"):ti,ab,kw OR ("Patient consultation"):ti,ab,kw 4192
#16 ("Patient contribution"):ti,ab,kw 13
#17 ("Community collaboration"):ti,ab,kw OR ("Community involvement"):ti,ab,kw OR ("Community consultation"):ti,ab,kw OR ("Community meeting"):ti,ab,kw OR ("Community mobilization"):ti,ab,kw 431
#18 #5 OR #6 OR #7 OR #8 OR #9 OR #10 OR #11 OR #12 OR #13 OR #14 OR #15 OR #16 OR #17 8834
#19 #18 AND #4 18

22 Scopus

(TITLE-ABS-KEY ("Mass drug administration") OR TITLE-ABS-KEY (mda) OR TITLE-ABS-KEY ("Mass chemoprophylaxis") OR TITLE-ABS-KEY ("Mass administration") OR TITLE-ABS-KEY ("Mass distribution") OR TITLE-ABS-KEY ("Mass treatment") OR TITLE-ABS-KEY ("Coordinated administration") OR TITLE-ABS-KEY ("Coordinated distribution") OR TITLE-ABS-KEY ("Coordinated treatment"))
AND
(TITLE-ABS-KEY ("Community-based intervention") OR TITLE-ABS-KEY ("participatory action Research") OR TITLE-ABS-KEY ("participatory research") OR TITLE-ABS-KEY ("participatory engagement") OR TITLE-ABS-KEY ("community research") OR TITLE-ABS-KEY ("action research") OR TITLE-ABS-KEY ("Community-Based Participatory Research") OR TITLE-ABS-KEY ("Community engagement") OR TITLE-ABS-KEY ("Community participation") OR TITLE-ABS-KEY ("Community partnership") OR TITLE-ABS-KEY ("Community collaboration") OR TITLE-ABS-KEY ("Community involvement") OR TITLE-ABS-KEY ("Community consultation") OR TITLE-ABS-KEY ("Community meeting") OR TITLE-ABS-KEY ("Community mobilization") OR TITLE-ABS-KEY ("Community empowerment") OR TITLE-ABS-KEY ("Community representative") OR TITLE-ABS-KEY ("Community input") OR TITLE-ABS-KEY ("Community led") OR TITLE-ABS-KEY ("Community driven") OR TITLE-ABS-KEY ("Stakeholder engagement") OR TITLE-ABS-KEY ("Stakeholder participation") OR TITLE-ABS-KEY ("Stakeholder partnership") OR TITLE-ABS-KEY ("Stakeholder collaboration") OR TITLE-ABS-KEY ("Stakeholder involvement") OR TITLE-ABS-KEY ("Stakeholder consultation") OR TITLE-ABS-KEY ("Stakeholder meeting") OR TITLE-ABS-KEY ("Public consultation") OR TITLE-ABS-KEY ("Public meeting") OR TITLE-ABS-KEY ("Public mobilization") OR TITLE-ABS-KEY (

"Public participation") OR TITLE-ABS-KEY ("User involvement") OR TITLE-ABS-KEY ("User participation") OR TITLE-ABS-KEY ("User contribution") OR TITLE-ABS-KEY ("Consumer participation") OR TITLE-ABS-KEY ("Consumer engagement") OR TITLE-ABS-KEY ("Consumer involvement") OR TITLE-ABS-KEY ("Consumer driven") OR TITLE-ABS-KEY ("Consumer consultation") OR TITLE-ABS-KEY ("Consumer empowerment") OR TITLE-ABS-KEY ("Citizen engagement") OR TITLE-ABS-KEY ("Citizen participation") OR TITLE-ABS-KEY ("Citizen involvement") OR TITLE-ABS-KEY ("Citizen deliberation") OR TITLE-ABS-KEY ("Citizen consultation") OR TITLE-ABS-KEY ("Citizen empowerment") OR TITLE-ABS-KEY ("Community action") OR TITLE-ABS-KEY ("Patient participation") OR TITLE-ABS-KEY ("Patient involvement") OR TITLE-ABS-KEY ("Patient consultation") OR TITLE-ABS-KEY ("Patient contribution"))

23 Global index medicus

(mh:(mass drug adminitration)) OR (tw:("mass drug adminitration")) OR (tw:(MDA)) OR (tw:("Mass chemoprophylaxis")) OR (tw:("Mass administration")) OR (tw:("Mass distribution")) OR (tw:("Mass treatment")) OR (tw:("Coordinated administration")) OR (tw:("Coordinated distribution")) OR (tw:("Coordinated treatment"))

AND

(mh:(Community-Based Participatory Research)) OR (mh:(Stakeholder Participation)) OR (mh:(Community Participation)) OR (tw:("Community-based intervention")) OR (tw:("participatory action Research")) OR (tw:("participatory research")) OR (tw:("participatory engagement")) OR (tw:("community research")) OR (tw:("action research")) OR (tw:("Community-Based Participatory Research")) OR (tw:("Community engagement")) OR (tw:("Community participation")) OR (tw:("Community partnership")) OR (tw:("Community collaboration")) OR (tw:("Community involvement")) OR (tw:("Community consultation")) OR (tw:("Community meeting")) OR (tw:("Community mobilization")) OR (tw:("Community empowerment")) OR (tw:("Community representative")) OR (tw:("Community input")) OR (tw:("Community led")) OR (tw:("Community driven")) OR (tw:("Stakeholder engagement")) OR (tw:("Stakeholder participation")) OR (tw:("Stakeholder partnership")) OR (tw:("Stakeholder collaboration")) OR (tw:("Stakeholder involvement")) OR

(tw:("Stakeholder consultation")) OR (tw:("Stakeholder meeting")) OR (tw:("Public consultation")) OR (tw:("Public meeting")) OR (tw:("Public mobilization")) OR (tw:("Public participation")) OR (tw:("User involvement")) OR (tw:("User participation")) OR (tw:("User contribution")) OR (tw:("Consumer participation")) OR (tw:("Consumer engagement")) OR (tw:("Consumer involvement")) OR (tw:("Consumer driven")) OR (tw:("Consumer consultation")) OR (tw:("Consumer empowerment")) OR (tw:("Citizen engagement")) OR (tw:("Citizen participation")) OR (tw:("Citizen involvement")) OR (tw:("Citizen deliberation")) OR (tw:("Citizen consultation")) OR (tw:("Citizen empowerment")) OR (tw:("Community action")) OR (tw:("Patient participation")) OR (tw:("Patient involvement")) OR (tw:("Patient consultation")) OR (tw:("Patient contribution"))

References

24

25 Balls-Berry JE, Acosta-Pérez E. The Use of Community Engaged Research Principles to Improve Health: Community Academic Partnerships for Research. P R Health Sci J. juin 2017;36(2):84-5.

26 Samuels AM, Odero NA, Odongo W, Otieno K, Were V, Shi YP, et al. Impact of Community-Based Mass Testing and Treatment on Malaria Infection Prevalence in a High-Transmission Area of Western Kenya: A Cluster Randomized Controlled Trial. Clin Infect Dis. 1 juin 2021;72(11):1927-35.

27 Odero NA, Samuels AM, Odongo W, Abong'o B, Gimnig J, Otieno K, et al. Community-based intermittent mass testing and treatment for malaria in an area of high transmission intensity, western Kenya: development of study site infrastructure and lessons learned. Malar J. déc 2019;18(1):255.

28 Adhikari B, James N, Newby G, Von Seidlein L, White NJ, Day NPJ, et al. Community engagement and population coverage in mass anti-malarial administrations: a systematic literature review. Malar J. déc 2016;15(1):523.

29 Chafer T, Majumdar MA, éditeurs. Routledge handbook of Francophone Africa. London New York: Routledge; 2024. 485 p.

30 Echenberg MJ. Africa in the time of cholera: a history of pandemics from 1817 to the present. New York: Cambridge University Press; 2011. 208 p. (African studies).

31 Tremblay M, Martin DH, Macaulay AC, Pluye P. Can we Build on Social Movement Theories to Develop and Improve Community-Based Participatory Research? A Framework Synthesis Review. Am J Community Psychol. juin 2017;59(3-4):333-62.

32 Clinical and Translational Science Awards Consortium: Principles of Community Engagement. NIH Publication No 11-7782. 2nd edition. 2011.

33 CIOMS: International Ethical Guidelines for Health-Related Research Involving Humans. 2016. Disponible sur: https://cioms.ch/wp-content/uploads/2017/01/WEB-CIOMS-EthicalGuidelines.pdf

34 Lavery JV. Building an evidence base for stakeholder engagement. Science. 10 août 2018;361(6402):554-6.

35 Aromataris E, Munn Z (Editors). JBI Manual for Evidence Synthesis. JBI, 2020.

36 TIDieR (Template for Intervention Description and Replication). Disponible sur: http://www.tidierguide.org/
