# Supplementary material for: Community engagement in mass drug administration participatory interventions: A scoping review
Source: PLoS Negl Trop Dis. 2025 Dec 1;19(12):e0013737. doi: 10.1371/journal.pntd.0013737 (PMC12742743; doi:10.1371/journal.pntd.0013737)
Supplement: S2 Text — (PDF) [file pntd.0013737.s002.pdf]

Appendix.
Full search strategy

	Community engagement	Mass drug administration
Free words	Community-based intervention*<br>participatory action Research<br>participatory research<br>participatory engagement*<br>community research<br>action research<br>Community-Based Participatory Research<br>Community engagement*<br>Community participation*<br>Community partnership<br>Community collaboration*<br>Community involvement*<br>Community consultation*<br>Community meeting*<br>Community mobilization*<br>Community empowerment<br>Community representative<br>Community input<br>Community led<br>Community driven<br>Stakeholder engagement*<br>Stakeholder participation*<br>Stakeholder partnership<br>Stakeholder collaboration*<br>Stakeholder involvement*<br>Stakeholder consultation*<br>Stakeholder meeting*<br>Public consultation*<br>Public meeting*<br>Public mobilization*<br>Public participation*<br>User involvement<br>User participation<br>User contribution<br>Consumer participation<br>Consumer engagement<br>Consumer involvement<br>Consumer driven<br>Consumer consultation<br>Consumer empowerment<br>Citizen engagement<br>Citizen participation	Mass drug administration*<br>MDA<br>Mass chemoprophylaxis<br>Mass administration*<br>Mass distribution*<br>Mass treatment*<br>Mass therap*<br>Coordinated administration*<br>Coordinated distribution*<br>Coordinated treatment*

	Citizen involvement<br>Citizen deliberation<br>Citizen consultation<br>Citizen empowerment<br>Community action*<br>Patient participation<br>Patient involvement<br>Patient consultation<br>Patient contribution	
Thesaurus /database	Medline :<br>Stakeholder participation<br>Community participation<br>Community-Based Participatory Research	Medline :<br>Mass drug administration
	Embase :<br>stakeholder engagement	Embase :<br>Mass drug administration
	Cochrane :<br>Stakeholder participation<br>Community participation	Cochrane :<br>Mass drug administration
	global index medicus :<br>Community-Based Participatory Research<br>Community Participation<br>Stakeholder Participation	global index medicus :<br>Mass drug administration

Pubmed equation :

"mass drug administration"[MeSH Terms] OR "mass drug administration"[Title/Abstract] OR "MDA"[Title/Abstract] OR "mass chemoprophylaxis"[Title/Abstract] OR "mass administration*"[Title/Abstract] OR "mass distribution*"[Title/Abstract] OR "mass treatment*"[Title/Abstract] OR "mass therap*"[Title/Abstract] OR "coordinated administration*"[Title/Abstract] OR "coordinated treatment*"[Title/Abstract] OR "coordinated distribution*"[Title/Abstract]
AND
"community participation"[MeSH Terms] OR "stakeholder participation"[MeSH Terms] OR "community based participatory research"[MeSH Terms] OR "community based intervention*"[Title/Abstract] OR "participatory action research"[Title/Abstract] OR "participatory research"[Title/Abstract] OR "participatory engagement*"[Title/Abstract] OR "community research"[Title/Abstract] OR "action research"[Title/Abstract] OR "community based participatory research"[Title/Abstract] OR "community engagement*"[Title/Abstract] OR "community participation*"[Title/Abstract] OR "community partnership*"[Title/Abstract] OR "community consultation*"[Title/Abstract] OR "community meeting*"[Title/Abstract] OR "community mobilisation*"[Title/Abstract] OR "community empowerment"[Title/Abstract] OR "stakeholder engagement*"[Title/Abstract] OR "stakeholder participation*"[Title/Abstract] OR "Stakeholder partnership"[Title/Abstract] OR "stakeholder collaboration*"[Title/Abstract] OR "stakeholder involvement*"[Title/Abstract] OR "stakeholder consultation*"[Title/Abstract] OR "stakeholder meeting*"[Title/Abstract] OR "public consultation*"[Title/Abstract] OR "public meeting*"[Title/Abstract] OR "public

mobilisation*"[Title/Abstract] OR "public participation*"[Title/Abstract] OR "local meeting*"[Title/Abstract] OR "Local empowerment"[Title/Abstract] OR "community action*"[Title/Abstract] OR "Community representative"[Title/Abstract] OR "Community input"[Title/Abstract] OR "Community led"[Title/Abstract] OR "Community driven"[Title/Abstract] OR "User involvement"[Title/Abstract] OR "User participation"[Title/Abstract] OR "User contribution"[Title/Abstract] OR "Consumer participation"[Title/Abstract] OR "Consumer engagement"[Title/Abstract] OR "Consumer involvement"[Title/Abstract] OR "Consumer driven"[Title/Abstract] OR "Consumer consultation"[Title/Abstract] OR "Consumer empowerment"[Title/Abstract] OR "Citizen engagement"[Title/Abstract] OR "Citizen participation"[Title/Abstract] OR "Citizen involvement"[Title/Abstract] OR "Citizen deliberation"[Title/Abstract] OR "Citizen consultation"[Title/Abstract] OR "Citizen empowerment"[Title/Abstract] OR "patient participation"[Title/Abstract] OR "patient involvement"[Title/Abstract] OR "patient consultation"[Title/Abstract] OR "patient contribution"[Title/Abstract]

EMBASE : 156 results
'mass drug administration'/exp OR 'mass drug administration':ab,ti OR 'mda':ab,ti OR 'mass chemoprophylaxis':ab,ti OR 'mass administration':ab,ti OR 'mass distribution':ab,ti OR 'mass treatment':ab,ti OR 'mass therap*':ab,ti OR 'coordinated administration':ab,ti OR 'coordinated distribution':ab,ti OR 'coordinated treatment':ab,ti
AND
'stakeholder engagement'/exp OR 'community-based intervention':ab,ti OR 'participatory action research':ab,ti OR 'participatory research':ab,ti OR 'participatory engagement':ab,ti OR 'community research':ab,ti OR 'action research':ab,ti OR 'community-based participatory research':ab,ti OR 'community engagement':ab,ti OR 'community partnership':ab,ti OR 'community participation':ab,ti OR 'community collaboration':ab,ti OR 'community involvement':ab,ti OR 'community consultation':ab,ti OR 'community meeting':ab,ti OR 'community mobilization':ab,ti OR 'community empowerment':ab,ti OR 'community representative':ab,ti OR 'community input':ab,ti OR 'community led':ab,ti OR 'community driven':ab,ti OR 'stakeholder engagement':ab,ti OR 'stakeholder participation':ab,ti OR 'stakeholder partnership':ab,ti OR 'stakeholder collaboration':ab,ti OR 'stakeholder involvement':ab,ti OR 'stakeholder consultation':ab,ti OR 'stakeholder meeting':ab,ti OR 'public consultation':ab,ti OR 'public meeting':ab,ti OR 'public mobilization':ab,ti OR 'public participation':ab,ti OR 'user involvement':ab,ti OR 'user participation':ab,ti OR 'user contribution':ab,ti OR 'consumer participation':ab,ti OR 'consumer engagement':ab,ti OR 'consumer involvement':ab,ti OR 'consumer driven':ab,ti OR 'consumer consultation':ab,ti OR 'consumer empowerment':ab,ti OR 'citizen engagement':ab,ti OR 'citizen participation':ab,ti OR 'citizen involvement':ab,ti OR 'citizen deliberation':ab,ti OR 'citizen consultation':ab,ti OR 'citizen empowerment':ab,ti OR 'community action':ab,ti OR 'patient participation':ab,ti OR 'patient involvement':ab,ti OR 'patient consultation':ab,ti OR 'patient contribution':ab,ti

WEB OF SCIENCE : 172 results
ALL=("mass drug administration") OR ALL=(MDA) OR ALL=("Mass chemoprophylaxis") OR ALL=("Mass administration") OR ALL=("Mass distribution") OR ALL=("Mass treatment") OR ALL=("Mass therap*") OR ALL=("Coordinated administration") OR ALL=("Coordinated distribution") OR ALL=("Coordinated treatment")

AND
ALL=("Community-based intervention") OR ALL=("participatory action Research") OR ALL=("participatory research") OR ALL=("participatory engagement") OR ALL=("community research") OR ALL=("action research") OR ALL=("Community-Based Participatory Research") OR ALL=("Community engagement") OR ALL=("Community participation") OR ALL=("Community partnership") OR ALL=("Community collaboration") OR ALL=("Community involvement") OR ALL=("Community consultation") OR ALL=("Community meeting") OR ALL=("Community mobilization") OR ALL=("Community empowerment") OR ALL=("Community representative") OR ALL=("Community input") OR ALL=("Community led") OR ALL=("Community driven") OR ALL=("Stakeholder engagement") OR ALL=("Stakeholder participation") OR ALL=("Stakeholder partnership") OR ALL=("Stakeholder collaboration") OR ALL=("Stakeholder involvement") OR ALL=("Stakeholder consultation") OR ALL=("Stakeholder meeting") OR ALL=("Public consultation") OR ALL=("Public meeting") OR ALL=("Public participation") OR ALL=("User involvement") OR ALL=("User participation") OR ALL=("User contribution ") OR ALL=("Consumer participation ") OR ALL=("Consumer engagement ") OR ALL=("Consumer involvement") OR ALL=("Consumer driven") OR ALL=("Consumer consultation") OR ALL=("Consumer empowerment") OR ALL=("Citizen engagement") OR ALL=("Citizen participation") OR ALL=("Citizen involvement") OR ALL=("Citizen deliberation") OR ALL=("Citizen consultation") OR ALL=("Citizen empowerment") OR ALL=("Community action") OR ALL=("Patient participation") OR ALL=("Patient involvement") OR ALL=("Patient consultation") OR ALL=("Patient contribution")

Cochrane library
Search Name:
Date Run: 05/01/2024 08:57:44
Comment:

ID Search Hits
#1 MeSH descriptor: [Mass Drug Administration] explode all trees 155
#2 ("mass drug administration"):ti,ab,kw OR ("MDA"):ti,ab,kw OR ("mass chemoprophylaxis"):ti,ab,kw OR ("mass administration"):ti,ab,kw OR ("mass distribution"):ti,ab,kw 4519
#3 ("Mass treatment"):ti,ab,kw OR ("coordinated administration"):ti,ab,kw OR ("coordinated distribution"):ti,ab,kw OR ("coordinated treatment"):ti,ab,kw 197
#4 #1 OR #2 OR #3 4683
#5 MeSH descriptor: [Stakeholder Participation] explode all trees 210
#6 MeSH descriptor: [Community Participation] explode all trees 2481
#7 ("Community-based intervention"):ti,ab,kw OR ("participatory action Research"):ti,ab,kw OR ("participatory research"):ti,ab,kw OR ("participatory engagement"):ti,ab,kw OR ("community research"):ti,ab,kw 1611
#8 ("action research"):ti,ab,kw OR ("Community-Based Participatory Research"):ti,ab,kw OR ("Community engagement"):ti,ab,kw OR ("Community participation"):ti,ab,kw OR ("Community partnership"):ti,ab,kw 2705
#9 ("Community empowerment"):ti,ab,kw OR ("Community representative"):ti,ab,kw OR ("Community input"):ti,ab,kw OR ("Community led"):ti,ab,kw OR ("Community driven"):ti,ab,kw 250

#10 ("Stakeholder engagement"):ti,ab,kw OR ("Stakeholder participation"):ti,ab,kw OR ("Stakeholder partnership"):ti,ab,kw OR ("Stakeholder collaboration"):ti,ab,kw OR ("Stakeholder involvement"):ti,ab,kw 322
#11 ("Stakeholder consultation"):ti,ab,kw OR ("Stakeholder meeting"):ti,ab,kw OR ("Public consultation"):ti,ab,kw OR ("Public meeting"):ti,ab,kw OR ("Public mobilization"):ti,ab,kw 37
#12 ("Public participation"):ti,ab,kw OR ("User involvement"):ti,ab,kw OR ("User participation"):ti,ab,kw OR ("User contribution"):ti,ab,kw OR ("Consumer participation"):ti,ab,kw 209
#13 ("Consumer engagement"):ti,ab,kw OR ("Consumer involvement"):ti,ab,kw OR ("Consumer driven"):ti,ab,kw OR ("Consumer consultation"):ti,ab,kw OR ("Consumer empowerment"):ti,ab,kw 73
#14 ("Citizen engagement"):ti,ab,kw OR ("Citizen participation"):ti,ab,kw OR ("Citizen involvement"):ti,ab,kw OR ("Citizen deliberation"):ti,ab,kw OR ("Citizen consultation"):ti,ab,kw 6
#15 ("Citizen empowerment"):ti,ab,kw OR ("Community action"):ti,ab,kw OR ("Patient participation"):ti,ab,kw OR ("Patient involvement"):ti,ab,kw OR ("Patient consultation"):ti,ab,kw 4192
#16 ("Patient contribution"):ti,ab,kw 13
#17 ("Community collaboration"):ti,ab,kw OR ("Community involvement"):ti,ab,kw OR ("Community consultation"):ti,ab,kw OR ("Community meeting"):ti,ab,kw OR ("Community mobilization"):ti,ab,kw 431
#18 #5 OR #6 OR #7 OR #8 OR #9 OR #10 OR #11 OR #12 OR #13 OR #14 OR #15 OR #16 OR #178834
#19 #18 AND #4 18

Scopus
(TITLE-ABS-KEY ("Mass drug administration") OR TITLE-ABS-KEY (mda) OR TITLE-ABS-KEY ("Mass chemoprophylaxis") OR TITLE-ABS-KEY ("Mass administration") OR TITLE-ABS-KEY ("Mass distribution") OR TITLE-ABS-KEY ("Mass treatment") OR TITLE-ABS-KEY ("Coordinated administration") OR TITLE-ABS-KEY ("Coordinated distribution") OR TITLE-ABS-KEY ("Coordinated treatment"))
AND
(TITLE-ABS-KEY ("Community-based intervention") OR TITLE-ABS-KEY ("participatory action Research") OR TITLE-ABS-KEY ("participatory research") OR TITLE-ABS-KEY ("participatory engagement") OR TITLE-ABS-KEY ("community research") OR TITLE-ABS-KEY ("action research") OR TITLE-ABS-KEY ("Community-Based Participatory Research") OR TITLE-ABS-KEY ("Community engagement") OR TITLE-ABS-KEY ("Community participation") OR TITLE-ABS-KEY ("Community partnership") OR TITLE-ABS-KEY ("Community collaboration") OR TITLE-ABS-KEY ("Community involvement") OR TITLE-ABS-KEY ("Community consultation") OR TITLE-ABS-KEY ("Community meeting") OR TITLE-ABS-KEY ("Community mobilization") OR TITLE-ABS-KEY ("Community empowerment") OR TITLE-ABS-KEY ("Community representative") OR TITLE-ABS-KEY ("Community input") OR TITLE-ABS-KEY ("Community led") OR TITLE-ABS-KEY ("Community driven") OR TITLE-ABS-KEY ("Stakeholder engagement") OR TITLE-ABS-KEY ("Stakeholder participation") OR TITLE-ABS-KEY ("Stakeholder partnership") OR TITLE-ABS-KEY ("Stakeholder collaboration") OR TITLE-ABS-

KEY ("Stakeholder involvement") OR TITLE-ABS-KEY ("Stakeholder consultation") OR TITLE-ABS-KEY ("Stakeholder meeting") OR TITLE-ABS-KEY ("Public consultation") OR TITLE-ABS-KEY ("Public meeting") OR TITLE-ABS-KEY ("Public mobilization") OR TITLE-ABS-KEY ("Public participation") OR TITLE-ABS-KEY ("User involvement") OR TITLE-ABS-KEY ("User participation") OR TITLE-ABS-KEY ("User contribution") OR TITLE-ABS-KEY ("Consumer participation") OR TITLE-ABS-KEY ("Consumer engagement") OR TITLE-ABS-KEY ("Consumer involvement") OR TITLE-ABS-KEY ("Consumer driven") OR TITLE-ABS-KEY ("Consumer consultation") OR TITLE-ABS-KEY ("Consumer empowerment") OR TITLE-ABS-KEY ("Citizen engagement") OR TITLE-ABS-KEY ("Citizen participation") OR TITLE-ABS-KEY ("Citizen involvement") OR TITLE-ABS-KEY ("Citizen deliberation") OR TITLE-ABS-KEY ("Citizen consultation") OR TITLE-ABS-KEY ("Citizen empowerment") OR TITLE-ABS-KEY ("Community action") OR TITLE-ABS-KEY ("Patient participation") OR TITLE-ABS-KEY ("Patient involvement") OR TITLE-ABS-KEY ("Patient consultation") OR TITLE-ABS-KEY ("Patient contribution"))

Global index medicus
(mh:(mass drug adminitration)) OR (tw:("mass drug adminitration")) OR (tw:(MDA)) OR (tw:("Mass chemoprophylaxis")) OR (tw:("Mass administration")) OR (tw:("Mass distribution")) OR (tw:("Mass treatment")) OR (tw:("Coordinated administration")) OR (tw:("Coordinated distribution")) OR (tw:("Coordinated treatment"))
AND
(mh:(Community-Based Participatory Research)) OR (mh:(Stakeholder Participation)) OR (mh:(Community Participation)) OR (tw:("Community-based intervention")) OR (tw:("participatory action Research")) OR (tw:("participatory research")) OR (tw:("participatory engagement")) OR (tw:("community research")) OR (tw:("action research")) OR (tw:("Community-Based Participatory Research")) OR (tw:("Community engagement")) OR (tw:("Community participation")) OR (tw:("Community partnership")) OR (tw:("Community collaboration")) OR (tw:("Community involvement")) OR (tw:("Community consultation")) OR (tw:("Community meeting")) OR (tw:("Community mobilization")) OR (tw:("Community empowerment")) OR (tw:("Community representative")) OR (tw:("Community input")) OR (tw:("Community led")) OR (tw:("Community driven")) OR (tw:("Stakeholder engagement")) OR (tw:("Stakeholder participation")) OR (tw:("Stakeholder partnership")) OR (tw:("Stakeholder collaboration")) OR (tw:("Stakeholder involvement")) OR (tw:("Stakeholder consultation")) OR (tw:("Stakeholder meeting")) OR (tw:("Public consultation")) OR (tw:("Public meeting")) OR (tw:("Public mobilization")) OR (tw:("Public participation")) OR (tw:("User involvement")) OR (tw:("User participation")) OR (tw:("User contribution")) OR (tw:("Consumer participation")) OR (tw:("Consumer engagement")) OR (tw:("Consumer involvement")) OR (tw:("Consumer driven")) OR (tw:("Consumer consultation")) OR (tw:("Consumer empowerment")) OR (tw:("Citizen engagement")) OR (tw:("Citizen participation")) OR (tw:("Citizen involvement")) OR (tw:("Citizen deliberation")) OR (tw:("Citizen consultation")) OR (tw:("Citizen empowerment")) OR (tw:("Community action")) OR (tw:("Patient participation")) OR (tw:("Patient involvement")) OR (tw:("Patient consultation")) OR (tw:("Patient contribution"))
