# Supplementary material for: Community engagement in mass drug administration participatory interventions: A scoping review
Source: PLoS Negl Trop Dis. 2025 Dec 1;19(12):e0013737. doi: 10.1371/journal.pntd.0013737 (PMC12742743; doi:10.1371/journal.pntd.0013737)
Supplement: Attachment — Submitted filename: ce.docx [file pntd.0013737.s005.docx]

Comments;

1. The author's definition of CE is to be better explained.

2. The advantages and disadvantages of CE are to be explained better.

3. The geographical variations in the CE different populations is to be stressed.

4. Why was COVID, a pandemic included in the article on mass prophylaxis? –please justify.
