# Supplementary material for: Community engagement in mass drug administration participatory interventions: A scoping review
Source: PLoS Negl Trop Dis. 2025 Dec 1;19(12):e0013737. doi: 10.1371/journal.pntd.0013737 (PMC12742743; doi:10.1371/journal.pntd.0013737)
Supplement: Attachment — Submitted filename: Response Anouk 19062025.docx [file pntd.0013737.s006.docx]

Journal Requirements:

1) Please ensure that the CRediT author contributions listed for every co-author are completed accurately and in full.
At this stage, the following Authors/Authors require contributions: Anouk Chouaïd. Please ensure that the full contributions of each author are acknowledged in the "Add/Edit/Remove Authors" section of our submission form.
The list of CRediT author contributions may be found here: <https://journals.plos.org/plosntds/s/authorship#loc-author-contributions>

We checked the CRediT author contributions which can be found after the conclusion, in the original submission.

2) We do not publish any copyright or trademark symbols that usually accompany proprietary names, eg ©,  ®, or TM  (e.g. next to drug or reagent names). Therefore please remove all instances of trademark/copyright symbols throughout the text, including:
- ® on page: 8.

We removed it.

3) Please upload all main figures as separate Figure files in .tif or .eps format. For more information about how to convert and format your figure files please see our guidelines: 
<https://journals.plos.org/plosntds/s/figures>

We did it

4) Please ensure that all Figure files have corresponding citations and legends within the manuscript. Currently, Figure 4 in your submission file inventory does not have an in-text citation. If the figure is no longer to be included as part of the submission, please remove it from the file inventory.

We did it

5) We have noticed that you have uploaded Supporting Information files, but you have not included a list of legends. Please add a full list of legends for your Supporting Information files after the references list.

We did it

6) In the online submission form, you indicated that "The data that support the findings of this study are available from the corresponding author, AC, upon request." All PLOS journals now require all data underlying the findings described in their manuscript to be freely available to other researchers, either
1. In a public repository
2. Within the manuscript itself
3. Uploaded as supplementary information.
This policy applies to all data except where public deposition would breach compliance with the protocol approved by your research ethics board. If your data cannot be made publicly available for ethical or legal reasons (e.g., public availability would compromise patient privacy), please explain your reasons by return email and your exemption request will be escalated to the editor for approval. Your exemption request will be handled independently and will not hold up the peer review process, but will need to be resolved should your manuscript be accepted for publication. One of the Editorial team will then be in touch if there are any issues.

The data is now available as a supplementary material.

Comments to the Authors:

Please note that one of the reviews is uploaded as an attachment.

Reviewers' Comments:

Reviewer's Responses to Questions

*Key Review Criteria Required for Acceptance?

As you describe the new analyses required for acceptance, please consider the following:


Methods

-Are the objectives of the study clearly articulated with a clear testable hypothesis stated?

-Is the study design appropriate to address the stated objectives?

-Is the population clearly described and appropriate for the hypothesis being tested?

-Is the sample size sufficient to ensure adequate power to address the hypothesis being tested?

-Were correct statistical analysis used to support conclusions?

-Are there concerns about ethical or regulatory requirements being met?*

Reviewer #2: Authors reported their study according to PRISMA guidelines and clearly detailed the selection process and exclusion criteria (figure 1). Author contribution is explicitly acknowledged throughout most of the methods section. Study design is justified and seems appropriate to address stated objectives.

Thank you for this nice feedback

Significant aspects are missing from the methods section:

- Definitions of CE and MDA used for the review remain unclear, compromising the understanding of the search strategy, inclusion of publications and data analysis - e.g., "We adopted a broad definition of CE" => please provide the definition and synonyms used
We agree that the definitions needed to be specified in the article. These terms are now defined in greater depth in the introduction and in the method. Moreover, the protocol and full search strategy were made available as supplementary material.

- Please provide a representative search string or synonyms used in your search strategy, for both semantic clusters (CE and MDA): this is essential for reproducibility of the study and should be provided in the manuscript, not only in the pre-registration.

We agree and the complete protocol and detailed research strategy have been added to the supplementary.

- Methods for data analysis are not described sufficiently in the manuscript, and the reference to the supplementary material is missing - e.g., "For data extraction and analysis, items from TIDIeR and MMAT checklist theoretical framework were used" => please explain which elements and the rationale for the choice of these elements, how you proceeded with their evaluation, and who conducted this work

Thank you for your comment. We added a short paragraph to detail and clarify the use of the tools.

Reviewer #3: objectives and methods reasonably met (see below for additional comments on the scope).

Thank you for this positive feedback.

*Results

-Does the analysis presented match the analysis plan?

-Are the results clearly and completely presented?

-Are the figures (Tables, Images) of sufficient quality for clarity?*

Reviewer #2: Overall, the results are well structured and match the stated objectives of the study.

Thank you for this positive feedback.

Points to improve include:
- Unfortunately, clear differences between MDA for NTDs and MDA for malaria are not acknowledged nor reflected in the results, which weakens the output of this publication.

- "Out of 24 interventions, 16 focused on NTDs" and among 8 malaria interventions, 4 belonged to the same research project: this is not reflected in the manuscript, which heavily focuses on malaria, including in the examples given in the results section.

We thank the reviewer for this comment. We have added a specific analysis comparing CE practices in malaria and NTD interventions, and clarified in the discussion that these differences likely reflect a temporal evolution in the conceptualization and implementation of community engagement.

- Meaning of reported numbers are unclear: do they refer to interventions or publications? E.g., "In most cases (N=30)". This is also valid for the following paragraphs and would be important to clarify.

Thank you for your comment. We clarified in the text whether we are talking about the intervention or the articles.

- Refer to supplementary materials for details on data analysis in the main text

Thank you for your comment. We included as supplementary materials :

1/ Complete protocol

2/ Detailed search strategy

3/ Detail on data analysis

Reviewer #3: Yes, but with comments.

*Conclusions

-Are the conclusions supported by the data presented?

-Are the limitations of analysis clearly described?

-Do the authors discuss how these data can be helpful to advance our understanding of the topic under study?

-Is public health relevance addressed?*

Reviewer #1: conclusions and discussion require changes.
the comment enclosed.

Reviewer #2: The discussion section is easy to read, clear and well-structured. Relevance of this study for public health is clearly stated and a list of recommendations to improve CE in MDA is provided and very helpful to improve further interventions (from "we emphasize the importance of combining" to "stability of funding, which is a key determinant of community participation"). You could emphasize further that this is a big output of your study. Limitations are stated, but should also include the focus on two languages only (French and English).

Thank you for this nice comment. We added the limitation you suggested.

Suggestion of further discussion points to improve the manuscript:
- CE is framed as a way of reaching marginalised groups/all sections of the population - but specific groups were systematically excluded from the analysed interventions ("pregnant women, sick people, children under age of two"), this would benefit from being discussed

These groups are essentially excluded for medical reasons, based on a safety principle and probably not a lack of community mobilization.

- In the results section, authors state that most studies do not evaluate the effect of CE on reduction of infection, which is the ultimate goal of MDA. The discussion would benefit from a greater analysis of this point: why is it so, what are the difficulties, what are the implications of not knowing the effect of CE?

It was a very useful comment that enriched the discussion. We added a paragraph regarding these points.

- Ethical aspects of CE could be addressed more: including the importance of CE (and information campaigns mentioned) for autonomy and informed consent in MDA; the importance of large coverage to reach reduction of infection and importance of this to make intervention ethically sound, balancing out the benefit/risk ratio for the uninfected majority. Refer to literature on MDA ethics.

Thank you for this comment. We add a specific paragraph on this topic on the discussion section.
- Please discuss the framing of "free clinics" and "providing drinking water" as incentives to participation as these are basic human needs (and essential to the roll-out of the intervention)

Thank you for your comment, we reworded this paragraph.

Reviewer #3: Yes, but with caveats mentioned below.

Editorial and Data Presentation Modifications?

Use this section for editorial suggestions as well as relatively minor modifications of existing data that would enhance clarity. If the only modifications needed are minor and/or editorial, you may wish to recommend “Minor Revision” or “Accept”.

Reviewer #2: - Figure 1 lacks arrows to guide reader through the selection pathway (might be an issue of formatting), also consider including "title-abstract screening" and "full-text screening" to make distinction between steps explicit

We corrected this error

- Figure 2 would be more readable and accurate as a bar chart

Thank you, we changed the type of figure.
- Figure 4 is not referred to in the main text

Thank you for this comment, we corrected it.

- Figures 3 and 4 could be merged which would increase the strength of the figure

Figure 4 replaces figure 3, which we are deleting.

- Figure 5 needs reviewing, the given information is not understandable at first glace: this information can either be provided in the text, or the figure should be transformed into a histogram or similar with readable percentages

We deleted figure 5 and provided the information in the text.

- A lot of abbreviations are introduced that are not used much and could be avoided to improve readability (e.g., DHA-PPQ, GMS etc.)

Thank you for this comment, we corrected it.

Summary and General Comments

 *Use this section to provide overall comments, discuss strengths/weaknesses of the study, novelty, significance, general execution and scholarship. You may also include additional comments for the author, including concerns about dual publication, research ethics, or publication ethics. If requesting major revision, please articulate the new experiments that are needed.*

Reviewer #2: This study is a scoping review about the use and contribution of community engagement in Mass Drug Administration (MDA) interventions. Authors focus on MDA conducted in the context of malaria and NTDs. Community engagement is a pivotal aspect to consider in MDA, both due to the ethical implications of delivering medication to people without prior diagnosis and due to the necessity of a large population coverage to reap the benefits of the measure. Moreover, the authors highlight a recent surge of publications on the topic of community engagement in MDA, which demands updated knowledge on these interventions. This research is highly needed to inform the design of MDA interventions. Authors present interesting results and a well-structured discussion that clearly articulates the relevance of results for Public Health and provides tangible recommendations.

Thank you for this positive feedback.

However, in its current form, the study presents significant shortcomings. Firstly, the rationale for the scope of the study is unclear. The authors include MDA interventions for NTDs and malaria, without explaining the rationale for analysing them jointly. Goals of MDA and methods used differ according to the disease (please refer to the WHO NTD roadmap 2021-2030, which outlines NTDs amenable to MDA, including levels of targeted control - e.g., control/ eliminate/eradicate, drugs of choice and target populations). Moreover, for many NTDs, MDA is a well-established public health measure, that has been carried out for decades in at-risk communities, whereas MDA for malaria seems to be carried out more frequently in research studies, is less common and not the primary strategy used to tackle the disease in at-risk settings. This pivotal difference is not acknowledged nor discussed in the manuscript. Additionally, most examples used throughout the manuscript focus on MDA for malaria, with MDA for NTDs being seldom addressed (which is questionable, both scientifically and given the scope of this journal) Clarifying the scope and justification for the selection of diseases and interventions is essential to improve the quality and output of this study. Authors could consider the following options:
- Clarify why malaria and NTDs are grouped in the analysis, as MDA has different goals for these diseases and implementation contexts: why can these interventions be compared? Why are other MDAs excluded (e.g., Mass Drug Administration of Azithromycin to Children under Five Years of Age to Promote Child Survival)?
- Separate the analysis of malaria MDA and NTD MDA and compare the results
- Focus the analysis on malaria and discuss results against examples of NTD MDA

Thank you for this constructive comment. To justify analyzing MDA interventions on NTDs and malaria together, we thoroughly reviewed the introduction and we compared the differences between the malaria and NTD interventions in the results section.

Other areas for general improvement include:

- Introduction structure and content: the introduction lacks a clear structure and flow, making it difficult to follow. Authors should reconsider and restructure the content of the introduction, to enable good understanding of the background and scope of this research. This links to the previous comment about the scoping of this review, which should be clearly explained in the introduction.

To clarify the plan and underline the points raised by your comment, we've deeply rewritten the introduction.

- Definitions: general definitions on which the research is based are missing. Both key concepts of the study, MDA and Community Engagement, are not clearly defined by the authors. Clarity on the definition used by the authors is essential to understand the study scope and methods (including the search strategy, making it reproducible) - this should be clarified early on in the introduction.
We added the definitions in the introduction and in the method section.

-A lot of sentences would benefit from being more specific, and key take-aways from the references should be made explicit - e.g., "Community-based participatory (CBP) intervention theory suggests that engaging community members as collaborators in the interventions to reduce health disparities is powerful on multiple levels" => please explain what levels

With the rewriting of the introduction, we aimed to be more precise in the terms used.

-Methods for data analysis should be detailed more, to enhance understanding and make the study reproducible

We now provide the research protocol and analysis framework in the appendix.

-Authors could consider discussing the ethical relevance of Community engagement in MDA in greater detail

We added this as a paragraph in the discussion.

Reviewer #3: Thank you for the opportunity to review the paper Community engagement in mass drug administration participatory interventions: a scoping review. The authors have evaluated how community engagement is done looking at this from the perspective of whether communities were “actively engaged”. MDA is an important tool for many diseases and coverage and equity are important factors, so this is an interesting topic and the last review was done nearly a decade ago.

Thank you for this positive feedback.

Major comments
The first key finding mentioned is: “Adopting participatory frameworks that promote shared decision-making and deeper community involvement is essential to improve outcomes and reduce inequalities.” Where is the evidence from the scoping review to support this statement? Or, is this based on other sources or opinions, in which case it is not really a finding from the review itself.

Yes, this sentence was confusing and we removed it from the revised version.

Have the authors used an appropriate strategy for identifying the literature? They find list only 24 interventions across 20 countries, and most are recent. A quick search on PubMed using the terms “Mass drug administration” & participation, returns 695 results. Many of which do not appear in the scoping review but appear to be relevant. E.g. 10.1179/000349804X3135 10.1179/136485906X105598 . The term community engagement was not very common before the year 2000 <https://pubmed.ncbi.nlm.nih.gov/?term=%22community+engagement%22&sort=date> . Moreover, all the papers that were reviewed in detail are from the published literature. But mass drug administrations have been used for disease control and elimination for many decades. For example, Ivermectin has been used billions of times as part of MDA campaigns. There must be a huge grey literature on this?

Indeed, me found that many articles use the terms “participation”, “community engagement”, “participatory research”, “community based”, but after reading the abstracts and full texts, only a limited number meet the inclusion criteria of the scoping review (figure 1). We put together a supplementary material containing the protocol, the complete search strategy and the data extraction grid. Furthermore, this research did not take grey literature into account. This has been added as a limitation in the limitations paragraph.

The article concludes: “The findings emphasize the importance of context-specific approaches, especially in addressing local sociocultural dynamics and including marginalized populations. Future interventions should prioritize sustainable capacity-building and adopt participatory frameworks that promote shared decision-making. Addressing these challenges could enhance the effectiveness of MDA campaigns and improve health outcomes in affected communities.” This is an easy statement to agree with, and the authors aren’t the first people to have noticed this. But if tailored, participatory engagement is not widely done or reported, why might this be? Is it always practicable in trial contexts, is it cost-effective in national programmes? Has it been tried but it actually doesn’t make as much difference in practice as it does in theory? What are the key steps? Are MDAs being done by teams without cultural knowledge of local communities, or often by people with excellent knowledge and experience of working with the communities they are seeking to persuade to participate in the MDA. Are there examples of participatory CE where it hasn’t worked or is it an unalloyed good?

A review of the literature provides little information to answer this question. The reasons are probably multi-factorial: difficulty of being cost-effective, need for long-term intervention, etc. Also, a lack of knowledge of the local context cannot be ruled out.

“Future interventions should prioritize sustainable, capacity-building approaches to maximize the effectiveness of CE in MDA efforts.” I agree, and expect most people who have been involved in conducting MDA would too. This scoping review appears to have been performed well and the findings are worth publishing, but the tone of the article is somewhat preachy and virtue-signalling, and this detracts from its impact. The point about participatory community engagement has been before, so I suggest the article would benefit from being toned down a bit.

We carefully reread the entire manuscript, and tried to adjust the tone as much as possible.

The Author Summary begins “The persistence of a public health approach that exploits community approaches is confirmed in the MDA field.” Please rephrase this, it is very hard to follow. Actually, it would be worth having a native English speaker proof read the whole article, as many of the sentence constructions are odd.

We had a native speaker proofread.

Apologies if these comments seem overly peevish. Again, this is a worthwhile review, properly done, with findings that will be of interest to a wide audience.

Thank you for this feedback, and for your review, which improved the manuscript considerably.

Figure resubmission:

While revising your submission, please upload your figure files to the Preflight Analysis and Conversion Engine (PACE) digital diagnostic tool, <https://pacev2.apexcovantage.com/>. PACE helps ensure that figures meet PLOS requirements. To use PACE, you must first register as a user. Registration is free. Then, login and navigate to the UPLOAD tab, where you will find detailed instructions on how to use the tool. If you encounter any issues or have any questions when using PACE, please email PLOS at [figures@plos.org](mailto:figures@plos.org). Please note that Supporting Information files do not need this step. If there are other versions of figure files still present in your submission file inventory at resubmission, please replace them with the PACE-processed versions.

Reproducibility:

To enhance the reproducibility of your results, we recommend that authors of applicable studies deposit laboratory protocols in [protocols.io](http://protocols.io), where a protocol can be assigned its own identifier (DOI) such that it can be cited independently in the future. Additionally, PLOS ONE offers an option to publish peer-reviewed clinical study protocols. Read more information on sharing protocols at <https://plos.org/protocols?utm_medium=editorial-email&utm_source=authorletters&utm_campaign=protocols>

Reviewer #1: conclusions and discussion require changes.

the comment enclosed. Comments;

1. The author's definition of CE is to be better explained.

Thank you for this comment. In the introduction and in the method section, we modified and refined the definition of CE.

2. The advantages and disadvantages of CE are to be explained better.

We discussed the advantages and disadvantages, both from an ethical and operational point of view.

3. The geographical variations in the CE different populations is to be stressed.

A paragraph in the results section specifies the geographical origin of the studies. However, within the framework of the protocol used, we had not planned to compare community involvement initiatives by geographical area

4. Why was COVID, a pandemic included in the article on mass prophylaxis? –please justif

It is true that this sentence was confusing. It has been removed.
