# Supplementary material for: Community engagement in mass drug administration participatory interventions: A scoping review
Source: PLoS Negl Trop Dis. 2025 Dec 1;19(12):e0013737. doi: 10.1371/journal.pntd.0013737 (PMC12742743; doi:10.1371/journal.pntd.0013737)
Supplement: Attachment — Submitted filename: Response R2 21082025.docx [file pntd.0013737.s007.docx]

We thank the reviewers for their careful reading of our manuscript and for their constructive comments, which have helped us improve the clarity and depth of our work. We have addressed each point raised, either by clarifying concepts, providing additional explanations, or highlighting relevant aspects in the text. Below, we provide a point-by-point response to all comments.

Anouk Chouaïd

Journal Requirements:

1) We have noticed that you have uploaded Supporting Information files, but you have not included a list of legends. Please add a full list of legends for your Supporting Information files after the references list.

The list of legend is after the reference list.

2) We note that your Data Availability Statement is currently as follows: "All data underlying the findings described in the manuscript is available either within the manuscript itself or in the documents provided as supplementary information.". Please confirm at this time whether or not your submission contains all raw data required to replicate the results of your study. Authors must share the “minimal data set” for their submission. PLOS defines the minimal data set to consist of the data required to replicate all study findings reported in the article, as well as related metadata and methods (https://journals.plos.org/plosone/s/data-availability#loc-minimal-data-set-definition).

For example, authors should submit the following data:

1) The values behind the means, standard deviations and other measures reported;

2) The values used to build graphs;

3) The points extracted from images for analysis..

Authors do not need to submit their entire data set if only a portion of the data was used in the reported study.

If your submission does not contain these data, please either upload them as Supporting Information files or deposit them to a stable, public repository and provide us with the relevant URLs, DOIs, or accession numbers. For a list of recommended repositories, please see https://journals.plos.org/plosone/s/recommended-repositories.

If there are ethical or legal restrictions on sharing a de-identified data set, please explain them in detail (e.g., data contain potentially sensitive information, data are owned by a third-party organization, etc.) and who has imposed them (e.g., an ethics committee). Please also provide contact information for a data access committee, ethics committee, or other institutional body to which data requests may be sent. If data are owned by a third party, please indicate how others may request data access.

All the date are shared as supplementary files.

3) As required by our policy on Data Availability, please ensure your manuscript or supplementary information includes the following:

- A numbered table of all studies identified in the literature search, including those that were excluded from the analyses.

- For every excluded study, the table should list the reason(s) for exclusion.

All excluded studies are shown in supplementary 4, with the reason for exclusion.

- A table of all data extracted from the primary research sources for the systematic review and/or meta-analysis. The table must include the following information for each study:

- Name of data extractors and date of data extraction

- Confirmation that the study was eligible to be included in the review.

- All data extracted from each study for the reported systematic review and/or meta-analysis that would be needed to replicate your analyses.

- If data or supporting information were obtained from another source (e.g. correspondence with the author of the original research article), please provide the source of data and dates on which the data/information were obtained by your research group.

- If applicable for your analysis, a table showing the completed risk of bias and quality/certainty assessments for each study or outcome. Please ensure this is provided for each domain or parameter assessed. For example, if you used the Cochrane risk-of-bias tool for randomized trials, provide answers to each of the signalling questions for each study. If you used GRADE to assess certainty of evidence, provide judgements about each of the quality of evidence factor. This should be provided for each outcome.

- An explanation of how missing data were handled.

This information can be included in the main text, supplementary information, or relevant data repository. Please note that providing these underlying data is a requirement for publication in this journal, and if these data are not provided your manuscript might be rejected.

All the required data are provided in the supplementary file n3

4) Kindly revise your competing statement to align with the journal's style guidelines: 'The authors declare that there are no competing interests.'

We did it.

Reviewers' comments:

Reviewer's Responses to Questions

Key Review Criteria Required for Acceptance?

As you describe the new analyses required for acceptance, please consider the following:

Methods

-Are the objectives of the study clearly articulated with a clear testable hypothesis stated?

-Is the study design appropriate to address the stated objectives?

-Is the population clearly described and appropriate for the hypothesis being tested?

-Is the sample size sufficient to ensure adequate power to address the hypothesis being tested?

-Were correct statistical analysis used to support conclusions?

-Are there concerns about ethical or regulatory requirements being met?

Reviewer #1: The objectives are clear

The study design is appropriate

Sample size is adequate.

Statistical test is fine.

No ethical consideration

Reviewer #2: (No Response)

Results

-Does the analysis presented match the analysis plan?

-Are the results clearly and completely presented?

-Are the figures (Tables, Images) of sufficient quality for clarity?

Reviewer #1: Analsis is appropriate.

Results are clear

Reviewer #2: (No Response)

Conclusions

-Are the conclusions supported by the data presented?

-Are the limitations of analysis clearly described?

-Do the authors discuss how these data can be helpful to advance our understanding of the topic under study?

-Is public health relevance addressed?

Reviewer #1: Conclusions are supported by data.

Limitations are stated.

The public healthrelevance is addressed.

Reviewer #2: (No Response)

Editorial and Data Presentation Modifications?

Use this section for editorial suggestions as well as relatively minor modifications of existing data that would enhance clarity. If the only modifications needed are minor and/or editorial, you may wish to recommend “Minor Revision” or “Accept”.

Reviewer #1: Accept

Reviewer #2:

- Figure 1: in the box "References removed", heading "other reason", the number is missing

Thank you, we corrected this.

- Results, under the section "Qualitative data also reported factors associated with participation", one bracket is missing and one sentence is double (same meaning twice): "Knowledge was often identified as a significant factor in determining whether an individual would take the drug. Knowledge about the disease and awareness of the intervention was associated with taking the drug."

Thank you, we corrected this.

Summary and General Comments

Use this section to provide overall comments, discuss strengths/weaknesses of the study, novelty, significance, general execution and scholarship. You may also include additional comments for the author, including concerns about dual publication, research ethics, or publication ethics. If requesting major revision, please articulate the new experiments that are needed.

Reviewer #1: The question raised in intial review has been addressed

Reviewer #2: The manuscript has significantly improved, especially the introduction and the discussion. The authors have well addressed the reviewers' comments.

A few minor suggestions for the authors' consideration:

- The article has a clear focus on malaria MDAs, as most publications selected were from this field and the examples taken in the results section are mostly malaria MDAs. Authors hint that this could be due to CE being most described in recent publications. The authors could clearly state and explain this focus on malaria in the introduction for more transparency with the reader.

Thank you for this suggestion. We intended to address this point in the introduction, with the sentence: “In this context, learning from past experiences of MDA to inform future interventions is crucial for ensuring ethical and effective action.” However, we agree that the novelty of CE in the context of malaria MDA deserves to be emphasized more clearly, and we will revise the text to make this explicit.

- It seems that CE is a relatively new practice or concept described in publications on MDA. Why is it the case? It would be interesting to see this discussed.

Thank you for this insightful comment. While CE practices have long been part of MDA, their explicit recognition and conceptual framing as “community engagement” in the literature are indeed relatively recent.

- The authors are strongly favorable to active participation of MDA target populations in intervention design and evaluation (rather than being passive information recipients). This seems to be ethically and socially sound, but seems to not have been done frequently. Why is it the case? What could be barriers to implementing active participation in MDA interventions and how could these be overcome?

Thank you for this comment. We agree that active participation of target populations in MDA remains limited, despite its ethical and social benefits. This may be due to practical challenges such as limited resources, time constraints, lack of training in participatory methods, or insufficient recognition of the value of community input. Overcoming these barriers likely requires capacity building, adequate funding, early engagement of communities, and integration of participatory approaches into routine MDA planning and evaluation.

We have added a sentence in the conclusion to highlight the importance of addressing barriers to active community participation in MDA interventions.

- In the discussion, authors state that “It would be interesting if malaria research teams interested in these evaluations could look at theory-based evaluation approaches or realist epistemology”. Please briefly explain the terms "theory-based evaluation approaches" and "realist epistemology" as these are not widely known.

Thank you for this comment. We add short definitions.
